# Characterization of Hydraulic Rock Diffusivity Using Oscillatory Pore Pressure

**DOI:** 10.1007/s11242-025-02176-2

**Published:** 2025-05-13

**Authors:** Dario Sciandra, Iman R. Kivi, Roman Y. Makhnenko, Dorothee Rebscher, Víctor Vilarrasa

**Affiliations:** 1 Global Change Research Group (GCRG), IMEDEA, CSIC-UIB, Esporles, Spain; 2 Laboratory of Soil Mechanics, École Polytechnique Fédérale de Lausanne, EPFL ENAC IIC LMS, Lausanne, Switzerland; 3 Institute of Environmental Assessment and Water Research, Spanish National Research Council (IDAEA-CSIC), Barcelona, Spain; 4 Department of Earth Science and Engineering, Imperial College London, London, UK; 5 Department of Civil & Environmental Engineering, University of Illinois at Urbana-Champaign, Urbana, IL USA; 6 Department of Earth and Environmental Sciences, California State University East Bay, Hayward, CA USA

**Keywords:** Analytical solutions, Permeability, Tight rock, Berea sandstone, Opalinus clay, Westerly granite

## Abstract

The interest of exploring deep geological resources for energy-related activities is rapidly increasing. Lowering the risks associated with these activities requires the development of fast and accurate in situ rock characterization methods. Monitoring and interpreting periodic signals, whether natural or man-induced, can provide valuable information about subsurface formations. This study focuses on improving the understanding of injection-induced pore pressure oscillations in confined formations and describes the use of periodic signals for characterizing hydraulic diffusivity. We revisit existing analytical solutions of cyclic pore pressure diffusion into geologic formations with one-dimensional or axisymmetric geometries and compare their performance with numerical simulations, including uncoupled hydraulic (H) and coupled hydro-mechanical (HM) models. We investigate the solutions in three main applications: (a) energy storage in porous rock, (b) CO₂-rich water injection into a caprock representative for CO_2_ storage, and (c) stimulation of an enhanced geothermal system in crystalline rock. The wave propagation extends over kilometer scales for the first case. In the second case, the wave propagation is confined to tens of centimeters. For the last case, the wave propagation occurs on the order of tens of meters. Numerical and analytical solutions match under identical assumptions, with errors of less than 3% across all the considered cases. While numerical solutions account for multidimensional hydro-mechanical rock response, analytical solutions provide an immediate initial approximation of the problem, enabling rapid reactions. This study highlights how simplified tools can aid in real-time interpretation for diverse subsurface energy applications, bridging analytical and numerical approaches for practical subsurface monitoring and characterization.

## Articles Highlights

- Analytical solutions can provide accurate estimates of the hydraulic diffusivity from interpretation of periodic signal.
- The attenuation length of oscillations spans orders of magnitude depending on the rock hydraulic diffusivity.
- Hydro-mechanical effects are non-negligible, especially as rock permeability decreases and stiffness increases.

## Introduction

Geo-energy technologies, such as subsurface energy storage, geological carbon storage, and geothermal energy, are becoming increasingly attractive in recent years owing to their key role in the green energy transition for mitigating climate change (IPCC 2022). These activities entail fluid injection into (or extraction from) the subsurface, which, in turn, perturb the initial equilibrium state of underground pore pressure and stress, giving rise to coupled hydro-mechanical (HM) effects. These effects include induced seismicity (Ge and Saar 2022), ground surface deformation (Rutqvist et al. 2010), and compromising caprock integrity (Vilarrasa 2014). Concerns around CO_2_ leakage through fine-grained caprocks (e.g., shales) should be addressed before the widespread deployment of geologic carbon storage (Kivi et al. 2022). In addition, the increasing number of perceivable and damaging earthquakes and changes to the hydraulic system induced by subsurface energy utilization have been negatively received by the public (Ellsworth 2013; Foulger et al. 2018). Proper HM characterization of the subsurface is essential to minimize the risks posed by these unsolicited side effects of geo-energy developments (Verdon 2014; Vilarrasa et al. 2013). However, the lack of clearly defined site characterization protocols, particularly in low-permeability rocks, necessitates the revision of existing characterization techniques, including HM coupled effects.

The spatial and temporal evolution of pore pressure and fluid flow in the subsurface during fluid injection or extraction is governed by hydraulic diffusivity *D* = *κ/S*_*s*_ where *κ* = *kρg*/*μ* is the hydraulic conductivity, *k* is the intrinsic permeability, *ρ* is the fluid density, *g* is the gravity acceleration, *μ* is fluid viscosity, and *S*_*s*_ is the specific storage coefficient. The higher the diffusivity, the faster the propagation of pressure perturbation through the rock (Ferris et al. 1962; Hantush 1964; Shih 2018). However, fluid flow in porous media is a coupled HM problem (Biot 1941; Cheng 2016; Cryer 1963). Pore pressure fluctuations give rise to changes in stress, which, in turn, cause the rock to deform and induce pore pressure alterations. A counterintuitive HM effect is injection-induced pressure drop in the adjacent low-permeability layers known as reverse water-level fluctuations (Hsieh 1996), which can only be reproduced by considering coupled HM effects (Blӧcher et al. 2008; Hsieh 1996; Slack et al. 2013; Vilarrasa et al. 2013; Wang 2000).

It is common practice in hydrogeology to derive diffusivity at the field scale from pumping tests (Ferris et al. 1962; Kruseman et al. 1970; Lohman 1972; Maliva 2016; Walton 2020). This technique is suitable for high-permeability aquifers, but unfeasible in low-permeability rock because fluid diffusion there takes much longer. By drilling a well that fully penetrates an aquifer and pumping fluid at a constant rate, the hydraulic properties can be derived from the pressure evolution at a distant observation well, e.g., by using the solutions for steady-state flow (Thiem 1906) or transient flow (Cooper Jr and Jacob 1946; Theis 1935). Alternatively, hydraulic diffusivity can be derived from periodic signal, not only in high-permeability rock, but also in tight rock (Hsieh et al. 1981).

Pressure fluctuations in groundwater of natural origin are ubiquitous, offering an opportunity to characterize rock, particularly if situated in the proximity of surface water bodies (i.e., a river, a lake, or an ocean). Ferris (1952) provided an analytical solution of the diffusion-type equation of tidal effects and applied it at the Ashland well station near Plate River, NE, USA, to determine the hydraulic properties of the rock. Bredehoeft (1967) developed a theory to estimate the specific storage coefficient of laterally extensive aquifers (exclusively vertical deformation), knowing their tidal response and Poisson’s ratio. These theories assume an incompressible solid matrix of the rock, and the same results can be inferred from the general three-dimensional theory of poroelasticity (Biot 1941). Hsieh et al. (1987, 1988) revised the theory and used it to calculate the transmissivity of the formation from the phase difference between tidal disturbances and water-level fluctuations in observation wells. Jiao and Tang (1999) modified Ferris’s traditional hydraulic solution, adding a term to account for potential leakage through confining layers, and applied the solution at the coastal aquifer in Chek Lap Kok Airport, Hong Kong.

The possibility of interpreting the oscillating pore pressure signal(s) to estimate the hydraulic properties of rock has been scrutinized in the laboratory (Bernabé et al. 2004; Candela et al. 2015; Faulkner and Rutter 2000; Hasanov et al. 2019; Kranz et al. 1990; Takahashi 2003). The experiments comprise imposing a harmonic pore pressure (or flow rate) excitation at one end of a cylindrical specimen while recording the fluctuating pressure response on the other end. Kranz et al. (1990) developed a hydraulic solution to the problem (assuming constant external stresses) and applied it to estimate the hydraulic diffusivity of Tennessee and Berea sandstones. Adachi and Detournay (1997) introduced a simplified HM coupling to Kranz’s solution to deal with the effect of varying axial stress in response to the oscillating pore pressure. Applying the solutions to oscillating pore pressure experiments at varying frequencies, Hasanov et al. (2020) highlighted a consistent discrepancy between the model predictions and measurements at relatively high frequencies (> 0.3 Hz). Analytical solutions derived to reproduce laboratory conditions may not be applicable to field-scale conditions.

**Fig. 1:**
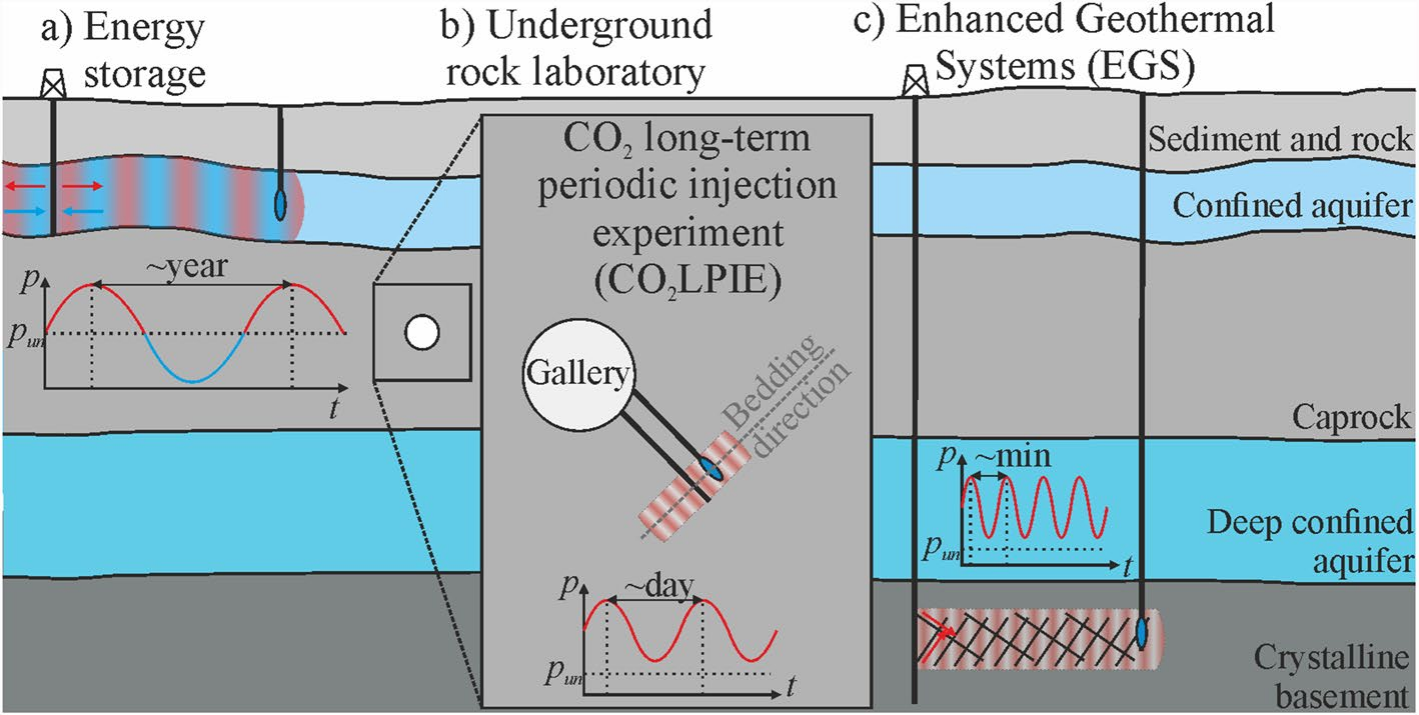
The three considered geo-energy applications are: **a** energy storage in shallow confined aquifers (~ 100–500-m depth), **b** CO_2_ Long-term Periodic Injection Experiment (CO_2_LPIE) at Mont Terri underground rock laboratory (~ 300-m depth), and **c** Enhanced Geothermal Systems (EGS) stimulation (> 4–6-km depth). The target rock formations are becoming deeper from left to right, while the injection period (or frequency) lowers from left to right

This study aims to provide an improved understanding of injection-induced pore pressure oscillations in confined formations and a description regarding the use of periodic signals for characterizing hydraulic diffusivity. We revisit existing analytical solutions of the problem of cyclic fluid injection into geologic formations of one-dimensional or axisymmetric geometries and compare their performance against numerical simulations, including uncoupled purely hydraulic (H) and fully-coupled HM simulations. We investigate the solutions in three main geo-energy applications: (a) energy storage in porous rock, (b) CO₂-rich water injection into a caprock representative for CO_2_ storage in an underground rock laboratory experiment, and (c) stimulation of an enhanced geothermal system in crystalline rock (Fig. 1). We choose as corresponding rock formations Berea sandstone, Opalinus Clay (shale), and Westerly granite, respectively, and assign a representative periodicity for each application.

## Methods

### The Diffusion Equation for Poroelastic Materials

Combining Darcy’s law with the fluid mass conservation equation leads to the pore pressure diffusion equation for a poroelastic material (Cheng 2016). Assuming an isotropic, homogeneous rock and constant fluid viscosity, the diffusion equation in terms of the pore pressure *p* and the mean stress *σ*_*m*_ = *σ*_*kk*_*/*3 can be written as (Biot 1941, 1956; Cheng 2016)

1 $$\frac{\partial p}{{\partial t}} - D\nabla^{2} p = \frac{B}{3}\frac{{\partial \sigma_{kk} }}{\partial t},$$

Here *D* = *κ/S*_*s*_ is the diffusion coefficient in which the specific storage can be expressed as *S*_*s*_ = [*ϕ*/*K*_*f*_ + 1/*K*–(1 + *ϕ*)/*K*_*s*_]*ρg*. *ϕ*, *K*_*f*_, *K*, and *K*_*s*_ denote, respectively, the effective rock porosity and bulk moduli of pore fluid, the rock skeleton under drained conditions, and the solid phase including isolated pores. The first invariant of the stress tensor *σ*_*kk*_ = *σ*_*xx*_ + *σ*_*yy*_ + *σ*_*zz*_ is taken positive under compression. Skempton’s *B* coefficient defines the pore pressure change as a result of changes in the mean stress under undrained conditions, i.e., *B* = Δ*p/σ*_*m*_ |_*un*_. The right-hand side of Eq. (1) accounts for mechanical coupling in the pore pressure diffusion model, meaning that it considers the stress and strain variation generated by the pressure propagation (Cheng 2016).

We analyze four analytical solutions for the diffusion equation with oscillating input pore pressure, under simplified flow and stress conditions. We consider 1D flow under constant stress assuming incompressible solid matrix, 1D and radial infinite flow under constant stress where the problem reduces to the traditional hydraulic diffusivity problem (stress and strain variations are neglected), and 1D flow under constant lateral stresses that accounts for a simplified HM coupling. For convenience, we report the results normalized by the characteristic length *λ*, which is a function of both the material properties (through the diffusivity *D*) and the pressure wave (through the frequency *ω*), *λ* = (*D/ω*)^1/2^.

### Uncoupled 1D Diffusion for Incompressible Solid Matrix

We consider first the analytical solution of a steady-periodic propagation of the pore pressure wave on an infinite-extent domain in the *x* direction, i.e., ∂^2^*p/*∂*y*^2^ = 0 and ∂^2^*p/*∂*z*^2^ = 0. The rock is subject to constant stress in all directions resulting in ∂*σ*_*kk*_*/*∂*t* = 0. Following Ferris (1952), if the solid phase is assumed incompressible, i.e., 1/*K*_*f*_ and 1/*K* >  > 1/*K*_*s*_, which is a common assumption in the context of soil mechanics, the hydraulic diffusivity *D* reduces to *D*_*s*_ and Eq. (1) to

2 $$\left\{ {\begin{array}{*{20}l} {\frac{\partial p}{{\partial t}} - D_{s} \frac{{\partial^{2} p}}{{\partial x^{2} }} = 0} \hfill \\ {D_{s} = \kappa \left( {\frac{\varphi }{{K_{f} }} + \frac{1}{K}} \right)^{ - 1} } \hfill \\ \end{array} } \right..$$

At the source (*x* = 0), we consider a sinusoidal pressure oscillation of constant amplitude *p*_0_ and frequency *ω* = 2π/*T*, where *T* is the period of the pressure wave, i.e., *p*(0, *t*) = *p*_0_ sin(*ωt*). Under these conditions, Jacob (1950) and Ferris (1952) derived an analytical solution for pore pressure amplitude as

3 $$A_{F} \left( x \right) = \frac{p(x)}{{p_{0} }} = e^{{ - x\sqrt {\frac{\omega }{{2D_{s} }}} }} = e^{{ - \frac{x}{\lambda }\sqrt {\frac{D}{{2D_{s} }}} }} ,$$

where the pore pressure amplitude ratio *A*_*F*_ decreases exponentially with distance from the source and as the square root of the frequency of the signal.

### Uncoupled 1D Diffusion of Oscillatory Pressure

In contrast with the assumption made by Jacob (1950) and Ferris (1952), the compressibility of the solid phase is non-negligible in rocks, oftentimes being on the same order of magnitude as its bulk compressibility (Cheng 2016). For this reason, we release the incompressibility assumption, meaning that the hydraulic diffusivity depends also on the compressibility of the solid phase and isolated pores (1/*K*_*s*_), while fluid flow and normal stress in the *x*-direction remain constant. Using these assumptions for a finite domain of length *L*, Kranz et al. (1990) developed an analytical solution by decomposing the pore pressure oscillation into amplitude *p*(*x*) and harmonic components, i.e., *p*(*x*, *t*) = *p*(*x*)·*e*^*iωt*^ with *p*(*x*) being a decreasing monotonic function of *x*. Imposing a constant amplitude and frequency at the source, the pore pressure amplitude as a function of distance is calculated as

4 $$\frac{p\left( x \right)}{{p_{0} }} = \frac{{\left[ {i\omega - S_{D} \left( {1 + i} \right)N} \right]e^{{\left( {1 + i} \right)N\left( {x - L} \right)}} - \left[ {i\omega + S_{D} \left( {1 + i} \right)N} \right]e^{{ - \left( {1 + i} \right)N\left( {x - L} \right)}} }}{{\left[ {i\omega - S_{D} \left( {1 + i} \right)N} \right]e^{{ - \left( {1 + i} \right)NL}} - \left[ {i\omega + S_{D} \left( {1 + i} \right)N} \right]e^{{\left( {1 + i} \right)NL}} }};$$

where *N* = (*ω*/2*D*)^1/2^, and *S*_*D*_ = *κπRK*_*f*_* /V*_*D*_ is a storativity term introduced by the laboratory setup, where *R* denotes the radius of the specimen cross-section, and *V*_*D*_ is the volume of the downstream compartment.

Considering an infinite domain (*L* → ∞) yields an expression for pore pressure diffusion at the field scale

5 $$A_{K} (x) = \left| {\frac{p\left( x \right)}{{p_{0} }}} \right| = \left| {e^{{ - x\left( {1 + i} \right)\sqrt {\frac{\omega }{2D}} }} } \right| = \left| {e^{{ - \left( {1 + i} \right)\frac{x}{\sqrt 2 \lambda }}} } \right|,$$

where | *p*(*x*)*/ p*_0_|= {[Re(*p*(*x*)*/ p*_0_)]^2^ + [Im(*p*(*x*)*/ p*_0_)]^2^} indicates the absolute value of the complex number. Equations (3) and (5) share similar exponential forms but with different hydraulic diffusivities: *D* as defined in Eq. (1) is larger than *D*_s_. Consequently, *A*_K_(*x*) ≥ *A*_F_(*x*), meaning that the amplitude diffuses further through materials with compressible solid constituents, while *A*_K_(*x*) solution coincides with *A*_F_(*x*) under the assumption of an incompressible solid matrix.

### One-Dimensional Diffusion of Oscillatory Input at Constant Lateral Stress

Previous simplified solutions consider constant stress (Sects. 2.2 and 2.3), uncoupling the pore pressure diffusion with the mechanical loading and deformation of the material, i.e., considering only the left-hand side of Eq. (1). Assuming constant lateral stress, i.e., ∂*σ*_*kk*_*/*∂*t* = ∂*σ*_*xx*_*/*∂*t*, the 1D diffusion equation becomes

6 $$\frac{\partial p}{{\partial t}} - D\frac{{\partial^{2} p}}{{\partial x^{2} }} = \frac{B}{3}\frac{{\partial \sigma_{xx} }}{\partial t}.$$

Adachi and Detournay (1997) solved this equation for a homogeneous elastic rock sample of length *L* and radius *R* under the same pore pressure oscillation function as in the work of *t* Kranz et al. (1990), i.e., *p*(0, *t*) = *p*_0_·*e*^*iωt*^ (Sect. 2.3). They assumed 1D flow in the *x*-direction and negligible shear stresses, acceptable for slender specimens of *R* <  < *L*. The solution is the superposition of the hydraulic and mechanical effects, with the former corresponding to Kranz’s solution as in Eq. (4) and the latter being

7 $$\left( {\frac{p\left( x \right)}{{p_{0} }}} \right)_{m} = \frac{B}{3}\frac{{\left( {1 - e^{{\left( {1 + i} \right)Nx}} } \right)\left[ {\frac{{S_{D} }}{D}\left( {1 - e^{{\left( {1 + i} \right)N\left( {x - 2L} \right)}} } \right) - \left( {1 + i} \right)N\left( {e^{{ - \left( {1 + i} \right)NL}} - e^{{\left( {1 + i} \right)N\left( {x - 2L} \right)}} - 1 + e^{{\left( {1 + i} \right)N\left( {x - L} \right)}} } \right)} \right]}}{{\frac{{S_{D} }}{D} + \left( {1 + i} \right)N + e^{{ - \left( {1 + i} \right)N2L}} \left[ {\frac{{S_{D} }}{D} - \left( {1 + i} \right)N} \right]}}.$$

For an infinite domain where *L* → ∞, the diffusivity equation becomes

8 $$\begin{aligned} A_{AD} (x) = & A_{K} (x) + A_{m} (x) = \left| {e^{{ - x\left( {1 + i} \right)\sqrt {\frac{\omega }{2D}} }} } \right| + \left| {\frac{B}{3}\left( {1 - e^{{ - x\left( {1 + i} \right)\sqrt {\frac{\omega }{2D}} }} } \right)} \right| \\ = & \left| {e^{{ - \left( {1 + i} \right)\frac{x}{\sqrt 2 \lambda }}} } \right| + \left| {\frac{B}{3}\left( {1 - e^{{ - \left( {1 + i} \right)\frac{x}{\sqrt 2 \lambda }}} } \right)} \right| \\ \end{aligned}$$

where *A*_*K*_(*x*) accounts for the pore pressure amplitude ratio under constant stresses (see Eq. (5)), and *A*_*m*_(*x*) represents the mechanical effect due to stress variation along the diffusion direction.

Comparing Eqs. (8) and (5), we notice that *A*_AD_(*x*) ≥ *A*_K_(*x*) since *A*_*m*_(*x*) ≥ 0. *A*_*m*_(*x*) is an increasing monotonic function of *x*, contrary to *A*_*K*_(*x*), meaning that the mechanical effect becomes more important at large distances, depending on the frequency of the signal.

### Uncoupled Two-Dimensional Diffusion of Periodic Pressure

In the previous sections, we assume 1D diffusion of pore pressure; however, for a vertical injection well (Fig. 1), the two-dimensional cylindrical diffusion should be considered. Assuming constant mean stress, resulting in ∂*σ*_*kk*_*/*∂*t* = 0, the pore pressure diffusion equation (Eq. 1) can be expressed in the cylindrical coordinates as

9 $$\frac{\partial p}{{\partial t}} - \frac{D}{r}\left[ {\frac{\partial }{\partial r}\left( {r\frac{\partial p}{{\partial r}}} \right) + \frac{\partial }{\partial \theta }\left( {\frac{1}{r}\frac{\partial p}{{\partial \theta }}} \right) + \frac{\partial }{\partial z}\left( {r\frac{\partial p}{{\partial z}}} \right)} \right] = 0$$

where *r* is the radial distance from the source, *z* is the location along the axis of symmetry (vertical), and *θ* is the angular coordinate. Considering a homogeneous isotropic material, i.e., ∂*p/*∂*z* = 0 and ∂*p/*∂*θ* = 0, and a stationarity pressure fluctuation *p*(*r*, *t*) = *p*(*r*)·*e*^*iωt*^, we obtain

10 $$Dr\frac{{d^{2} p\left( r \right)}}{{dr^{2} }} + D\frac{dp\left( r \right)}{{dr}} - i\omega \,r \cdot p\left( r \right) = 0.$$

For an infinite domain, the solution of Eq. (10) is a linear combination of two Bessel functions of zero order (Olver and Maximon 2010)

11 $$p\left( r \right) = a \cdot I_{0} \left( {\sqrt i r\lambda^{ - 1} } \right) + b \cdot K_{0} \left( {\sqrt i r\lambda^{ - 1} } \right),$$

where *I*_0_ is a hyperbolic Bessel function of the first kind of order zero, and *K*_0_ is a hyperbolic Bessel function of the second kind of order zero calculated for the complex number (*i*)^1/2^*r/λ*, and *a* and *b* are constant values to be defined from boundary conditions. The same theory was employed by Carslaw and Jaeger (1959) to address the heat conduction problem.

We consider an injection well of radius *r*_0_ and no far-field pressure variation as *r* approaches infinity, i.e., *p*(*r*_0_) = *p*_0_ and *p*(*r* → ∞) = 0, respectively. Under these conditions, the first term in Eq. (11) can be neglected as *a* = 0 and *b* corresponds to *p*_0_/*K*_0_((*i*)^1/2^*r*_0_*/λ*). The amplitude attenuation with distance is thus governed by

12 $$A_{R} = \tfrac{p\left( r \right)}{{p_{0} }} = \frac{{K_{0} \left( {\sqrt i r\lambda^{ - 1} } \right)}}{{K_{0} \left( {\sqrt i r_{0} \lambda^{ - 1} } \right)}}$$

## Test Cases and Numerical Models

### Material Properties

We apply the analytical solutions of pore pressure diffusion to three geo-energy applications that involve oscillating pore pressure with different periods: (a) energy storage in porous rock, (b) caprock integrity during periodic CO_2_ injection—the CO_2_LPIE experiment (Rebscher et al. 2020; Sciandra et al. 2022a, 2022b), and (c) enhanced geothermal system stimulation (Fig. 1).

For the energy storage, we assign a period of 1 year, following the annual fluctuations in energy demand and production, as happens in natural gas and hydrogen storage (e.g., Lysyy et al. 2021). In the CO_2_LPIE experiment, we assign a period of 1 week, which permits observation of periodic pore pressure variation in shale at distances in the order of tens of centimeters. Considering the stimulation of an enhanced geothermal system, we assign a short period, in the order of hours—typical of the so-called cyclic or soft stimulation (Hofmann et al. 2018, 2019). In this way, we cover a wide range of both rock diffusivity and the period of the signal.

We select the parameter referring to three well-characterized rocks as representative hosts for these applications: Berea sandstone for energy storage (Makhnenko and Labuz 2016), Opalinus Clay (shale) from Mont Terri Lab as a representative caprock for CO_2_ storage (Makhnenko et al. 2017; Makhnenko and Podladchikov 2018), and Westerly granite for enhanced geothermal system stimulation (Nur and Byerlee 1971). The properties controlling the hydraulic conductivity and storage coefficient are representative of stresses associated with 200 m for Berea sandstone, 300 m for Opalinus Clay, and 1000 m for Westerly granite (Table 1).

**Table 1:** Hydro-mechanical properties of the rock types considered in this study

Property	Berea sandstone^1^ (*T* = 365 d)	Opalinus Clay^2,3^ (*T* = 7 d)	Westerly granite^4^ (*T* = 0.5 d)
*K* (GPa)	6.7	1.9	25.0
$K_{s}$(GPa)	30.0	8.1	45.4
*K*_*f*_ (GPa)	2.25	2.25	2.25
*ϕ* (-)	0.23	0.12	0.01
*k* (m^2^)	1.9·10^–13^	2.0·10^–20^	4.0·10^–19^
*B* (-)	0.80	0.90	0.81
*D*_*s*_ (m^2^/s)	0.76	3.45·10^–8^	9.00·10^–6^
*D* (m^2^/s)	0.90	4.53·10^–8^	1.80·10^–5^
*λ* (m)	2.13·10^3^	6.60·10^–2^	3.52·10^–1^

^1^Makhnenko and Labuz (2016);^2^Makhnenko et al. (2017); ^3^Makhnenko and Podladchikov (2018); ^4^Nur and Byerlee (1971)

We assume that the injected fluid for storage and stimulation purposes can be represented by water, which has a compressibility 1/*K*_*f*_ of around 0.44 GPa^−1^ and a viscosity of *μ*  = 0.001 Pa·s at room temperature (Cheng 2016). The host rock for CO_2_LPIE is Opalinus Clay, a shale characterized by a low diffusivity and considered as a representative caprock for CO_2_ storage. The experiment aims at injecting gaseous CO_2_ at a mean overpressure of 1 MPa, with an amplitude on the order of 0.1 MPa. Preliminary simulation results show that the high capillary-entry pressure of Opalinus Clay (⁓ MPa) prevents free-phase CO_2_ intrusion into the pore network, while the periodic pore pressure signal propagates much ahead of the diffusive aqueous CO_2_ front (Sciandra et al. 2022a; 2022b). Therefore, we also assume water flow for this case. Given the reported hydro-mechanical properties, we calculate that the hydraulic diffusivities *D* and *D*_*s*_ vary by several orders of magnitude among the three materials. The effect of solid-phase compressibility on diffusion coefficient, i.e., the difference between *D* and *D*_*S*_, is the most pronounced for Westerly granite (a factor of two), since the rock has a large bulk modulus *K* and small porosity *ϕ*, decreasing the contribution of rock and fluid on the storage coefficient. Finally, combining the representative material and signal properties, we derive the characteristic length *λ* (Table 1).

### Geometry Definition

We assess the applicability and accuracy of the analytical solutions presented through comparison with numerical simulations. We solve the problem for two different geometries using the fully-coupled finite-element code CODE_BRIGHT (Olivella et al. 1994, 1996). The linear flow model is compared with the uncoupled 1D diffusion of oscillatory pressure, for the case of both compressible and incompressible solid matrices *A*_K_ and *A*_F_ (Sects. 2.3 and 2.2, respectively) and the 1D diffusion under constant lateral stress *A*_AD_ (Sect. 2.4). The axisymmetric solution is compared with the uncoupled radial diffusion of periodic pressure perturbation *A*_R_ (Sect. 2.5).

**Fig. 2:**
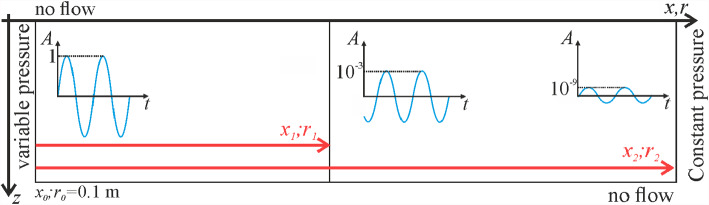
Geometry and boundary conditions of the hydraulic model. The sketch represents both the 1D and the radial case, where for the latter, the symmetry around *r* = 0 is assumed

**Fig. 3:**
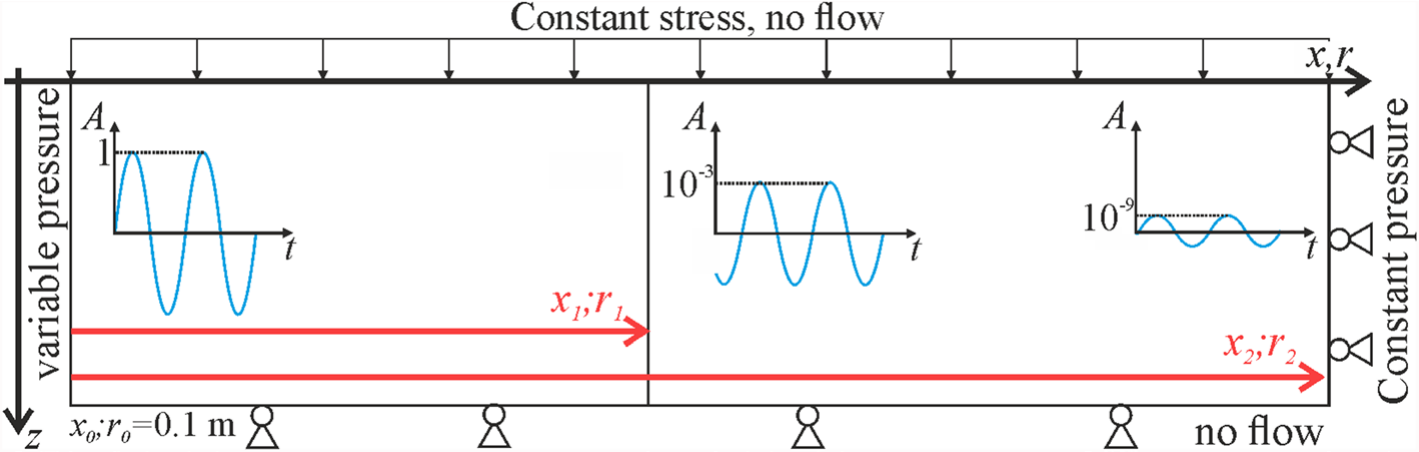
Geometry and boundary conditions of the coupled hydro-mechanical model for both the 1D and the radial case where the symmetry around *r* = 0 is assumed

In all simulations, the hydraulic diffusion coefficient *D* is employed as given in Table 1. In addition to considering the compressibility of the solid matrix, *D* is used for almost all the analytical solutions reported here, leaving *D*_*s*_ to Ferris' formulation alone (Sect. 2.2), which has been previously validated for in situ conditions (Ferris 1952). We define two different areas: one from the injection source on the left boundary to *x*_1_ (and *r*_1_), hereinafter also referred to as the *monitoring region*. The other area includes an extension of the model geometry to a distance of *x*_2_ (and *r*_2_), to avoid boundary effects (Figs. 2 and 3). To evaluate these distances, we rearrange Eq. (5) into

13 $$x_{j} = - \sqrt {\frac{2D}{\omega }} \cdot \ln \left( {A_{j} } \right) = - \sqrt 2 \lambda \cdot \ln \left( {A_{j} } \right)\quad {\text{with}}\;j = 1,2$$

We define *x*_*1*_ (and *r*_*1*_) and *x*_*2*_ (and *r*_*2*_), as distances at which amplitude ratios attenuate to *A*_*1*_ = 10^–3^ and *A*_*2*_ = 10^–9^, respectively. This implies that for an initial amplitude *p*_0_ in the order of MPa, the minimum amplitude recorded across the monitoring region will be on the order of kPa. The criterion for the definition of *A*_*2*_ considers the precision limits of the numerical simulations. We adopt the same distances for the cylindrical geometry for the sake of comparison with the 1D geometry. The calculated values of the boundary locations for the three geo-energy applications subject to representative pressure fluctuation periods are listed in Table 2.

**Table 2:** Length of the domain (see Fig. 2), evaluated with Eq. (13) for amplitude attenuations of 10^–3^ and 10^–9^, respectively

Distances	Berea sandstone (*T* = 365 d)	Opalinus Clay (*T* = 7 d)	Westerly granite (*T* = 0.5 d)
*x*_*1*_; *r*_*1*_ (m)	2.27·10^4^	0.65	3.4
*x*_*2*_; *r*_*2*_ (m)	6.80·10^4^	1.92	10.3

### Hydraulic Model

For both 1D and axisymmetric geometries, we assume horizontal flow, neglecting the effect of gravity forces. In addition, we impose no flow on the top and bottom boundaries and constant pressure on the right boundary. At the left boundary, we impose a sinusoidal variation of the pore pressure with constant amplitude *p*_0_ and frequency *ω* (Fig. 2). Finally, we numerically solve Eq. (1) with the diffusivity coefficient *D* and the right-hand side dealing with stress changes set to zero. Throughout this paper, we refer to this model as hydraulic and accordingly, the obtained pressure amplitude as *A*_H_, since mechanical constraints on pressure diffusion are not considered, although the reservoir stiffness is accounted for through the storage term *S*_s_ introduced in the diffusivity equation.

### Hydro-Mechanical Model

We assume a homogeneous isotropic poroelastic material fully saturated with water. We solve the mass conservation of water and Darcy’s law together with linear momentum balance and poroelastic constitutive equations of the porous medium in a fully-coupled manner. The mechanical boundary conditions include zero displacements perpendicular to lateral and bottom boundaries and a uniformly distributed stress on the upper one (Fig. 3). These boundary conditions, which are representative of the HM problem at the field scale, differ from those considered by Adachi and Detournay (1997), who assumed no displacement in the upper and lower boundaries and constant stress on the right boundary (Sect. 2.4), representative of laboratory experiments in a core holder. We refer to this model as hydro-mechanical and the obtained pressure amplitude as *A*_HM_.

## Results

### Comparison Between Analytical and Numerical Solutions

**Fig. 4:**
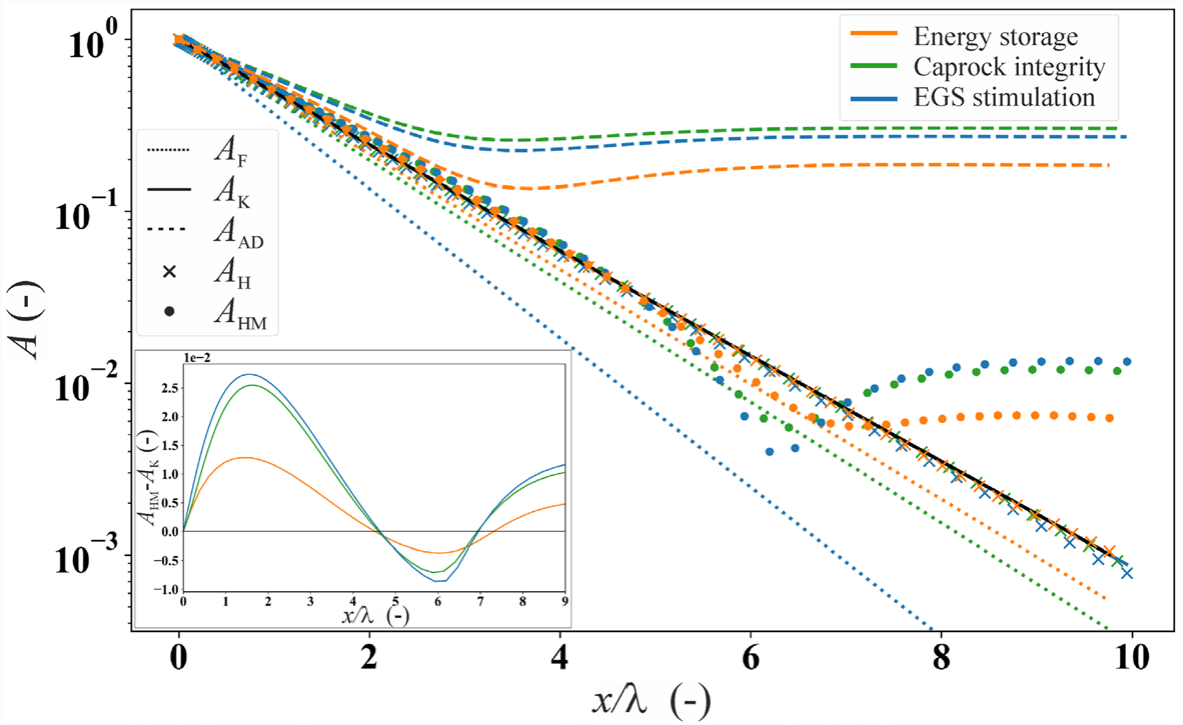
Amplitude ratio *A* as a function of linear distance from the well, normalized by the characteristic length *λ*, for the three examined case studies (see Fig. 1). Dashed and continuous lines represent the analytical solutions, while the symbols represent the numerical solutions. In the inset, we plot the difference between the HM and Kranz’s (H) solutions, *A*_HM_-*A*_*K*_, for all three case studies

**Fig. 5:**
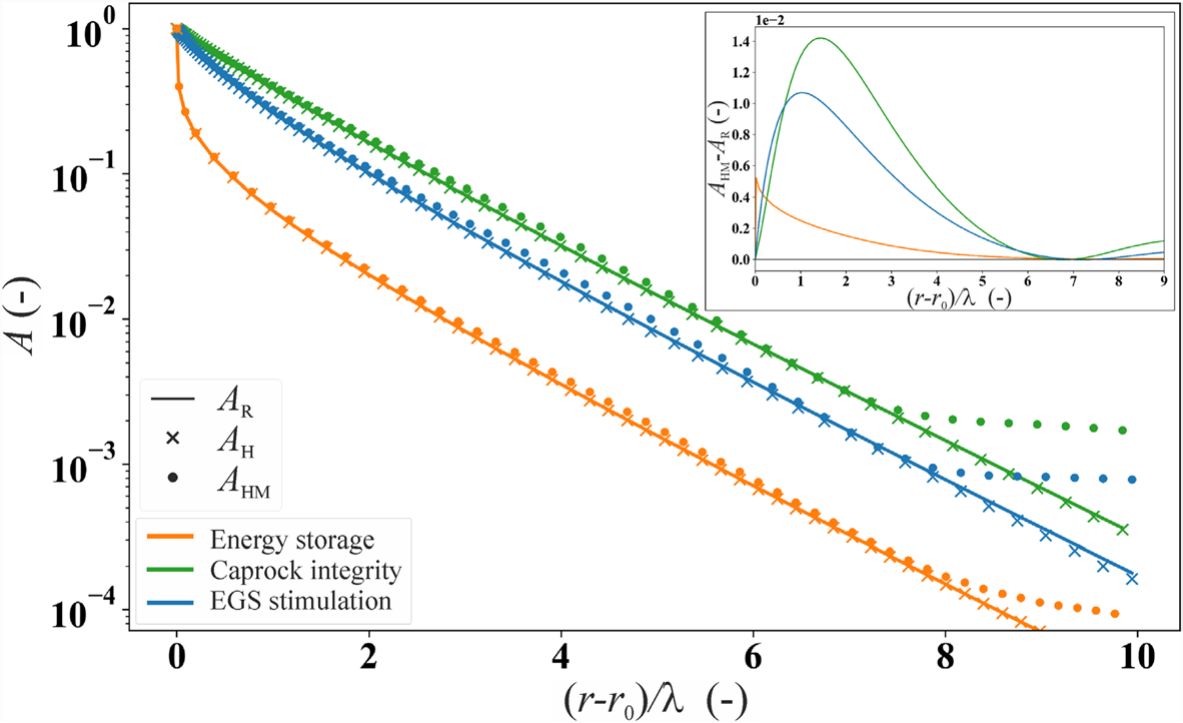
Amplitude ratio *A* as a function of the dimensionless radial distance from the well, normalized by the characteristic length *λ*, for the three examined case studies (see Fig. 1). The lines represent the analytical solutions while the symbols represent the numerical solutions. In the inset, we plot the difference between *A*_HM_-*A*_*R*_, for all three case studies

Figures 4 and 5 show the propagation of pressure in confined geological layers of infinite extent. We explore two flow geometries: linear (Fig. 4) and cylindrical (Fig. 5). The results are presented in terms of the dimensionless attenuation of the injection/extraction wave amplitude, obtained for the various theories and case studies detailed in Sect. 3. To facilitate comparison across different scenarios, we normalize the distance from the source using the characteristic length *λ* (refer to Table 1). This normalization allows us to present all results in a unified plot.

The analytical solutions *A*_*F*_, *A*_*K*_, and the numerical solution *A*_*H*_ for the one-dimensional diffusion assumption exhibit linear trends in the semi-logarithmic plot (Fig. 4). This linear behavior arises due to the constant value of the specific storage *S*_*s*_ in the three solutions, if wave propagation-induced deformations are neglected. Specifically, for a fixed characteristic length *λ*, the solutions *A*_*K*_ and *A*_H_ (the black line and crosses, respectively, in Fig. 4) coincide with each other for all three scenarios, representing a perfect match between the analytical and numerical solutions for the purely hydraulic problem.

*A*_F_ curves (dotted lines in Fig. 4) exhibit for all scenarios a steeper slope compared to *A*_K_ (and *A*_*H*_), indicating a greater attenuation of the wave. This increased attenuation is attributed to the absence of compression/expansion of the solid constituents caused by the injection/extraction in the *A*_F_ model. While such an assumption may be acceptable for shallow or unconsolidated sediments where the bulk modulus of the soil can be much smaller than that of solid constituents, it appears inadequate for consolidated rock (Cheng 2016). This behavior is more evident for enhanced geothermal systems stimulation than for both the CO_2_LPIE and energy storage cases because of the lower Biot‘s coefficient *α* (higher *K*/*K*_s_ ratio) of the stiff Westerly granite, approximately equal to 0.44, compared to the 0.76 and 0.78 for Opalinus Clay and Berea sandstone, respectively (Table 1).

The analytical solution *A*_AD_ (dashed lines in Fig. 4) and the numerical solution *A*_HM_ (dots in Fig. 4) include the instantaneous deformation of the rock induced by pore pressure wave propagation. In all scenarios, both solutions indicate a nonlinear behavior of the amplitude ratio that approaches a constant value at some distance from the source. The *A*_HM_ considers vertical deformation, while the simplified analytical solution *A*_AD_ accounts solely for lateral deformation. Considering *A*_HM_ with boundary conditions being more representative of the in situ conditions as the reference scenario, we find that *A*_AD_ yields a significant overestimation of the amplitude ratio (Fig. 4). Yet, the accuracy of the *A*_AD_ solution for its simplifying assumptions is verified through comparison with numerical solutions with identical boundary conditions (see Appendix 1).

A comparison between hydraulic solutions *A*_H_ and *A*_K_ and the hydro-mechanical solution *A*_HM_ (see inset in Fig. 4) reveals that the difference between the two couplings follows a specific pattern. Initially, this difference increases with distance from the source until it reaches a peak (around *x*/*λ* = 2). After that value, it decreases, becoming negative when x/*λ* approaches 4.5. Finally, increases again, converging to a positive value. Additionally, we note that the magnitude of *A*_HM_-*A*_*K*_ remains limited to 10^–2^, i.e., 1%. This difference is less pronounced in the energy storage scenario compared to the CO_2_LPIE and enhanced geothermal systems stimulation cases.

In the context of cylindrical pore pressure diffusion (Fig. 5), determining the dimensionless distance requires subtracting the radius of the cylindrical source *r*_0_, before dividing by the characteristic length *λ*. Similar to the linear geometry case, the analytical solution *A*_R_ is in good agreement with the numerical solution *A*_H_ for the cylindrical geometry. The amplitude attenuation is higher compared with the linear geometry (Fig. 4), when all other conditions remain constant, as the cylindrical flow provides more space for the pressure to diffuse. Specifically, the analytical solution *A*_R_ exhibits a nonlinear attenuation trend for values of the radial dimensionless distance smaller than 2 (Fig. 5). This decrease is more pronounced in the energy storage application, moderately visible in enhanced geothermal systems stimulation, and almost negligible for CO_2_LPIE, exhibiting an inverse proportionality to rock diffusivity *D* (Table 1). Notably, the periodicity does not impact this behavior, which is solely governed by the rock properties. Conversely, for values of the dimensionless distance larger than 2, the curves exhibit a linear decrease in a semi-logarithmic plot (Fig. 5).

A comparison between the hydro-mechanical solution *A*_HM_, achieved numerically, and hydraulic solutions *A*_H_ and *A*_R_ for the cylindrical geometry shows that the deformation of the reservoir affects the results for values of (*r*–*r*_0_)/*λ* > 7, leading to a significant divergence between the two couplings (Fig. 5). However, when observing the difference *A*_HM_-*A*_*R*_ (inset in Fig. 5), we note that the disparity is confined to 10^–2^, i.e., 1%. This follows a similar behavior as analyzed for the one-dimensional case (inset in Fig. 4), albeit with lower absolute values for each case.

### Attenuation for Various Analytical Solutions

**Fig. 6:**
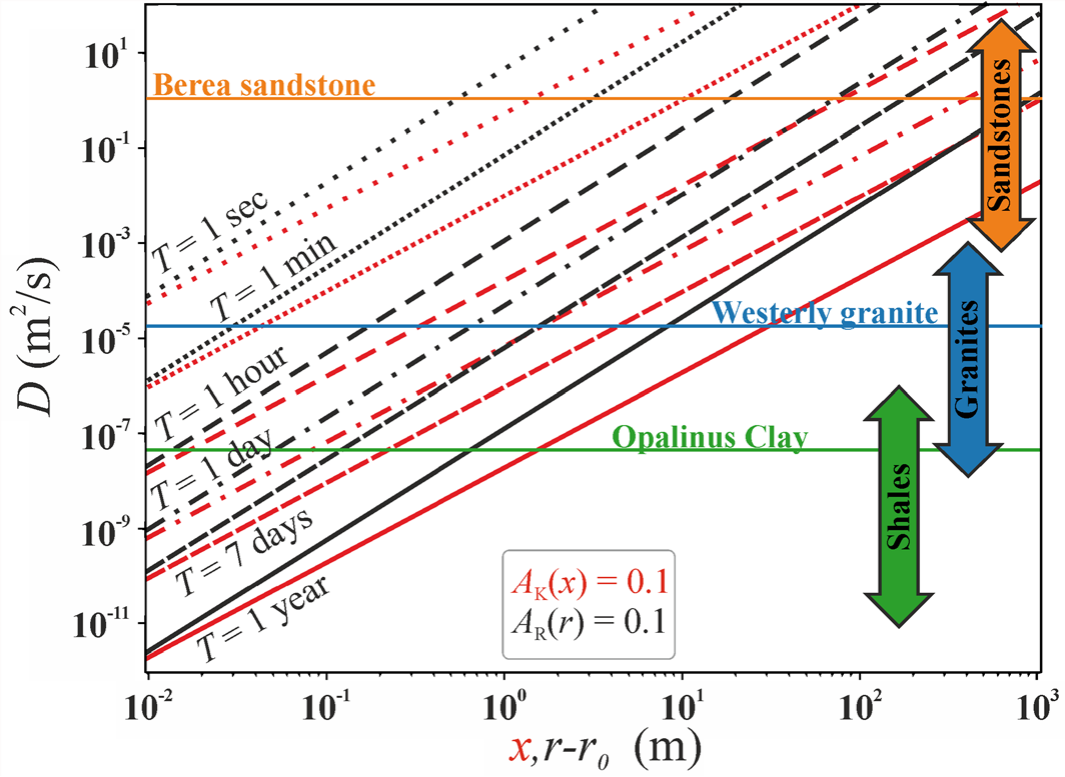
Variation of threshold values for the attenuation ratio *A*_K_ = 0.1 (red lines) and *A*_R_ = 0.1 (black lines), as a function of the hydraulic diffusivity *D* for different periods *T*. The orange, blue, and green lines refer to *D* values for the considered geo-energy applications (Sect. 3), while the ranges of *D* for different rock types (sandstone, granites, and shales) are based on hydraulic data from Brace (1980)

Following the comparison of the analytical solutions (*A*_K_ and *A*_R_) with the numerical solution (*A*_HM_, provided for both geometries), we observe that the difference of amplitude ratio remains limited to around 1% for both linear and cylindrical flow cases (insets in Fig. 4 and Fig. 5, respectively). Consequently, we deem it reasonable to establish a threshold for the amplitude ratio at 10% or one order of magnitude higher (Fig. 6), to compare the extent to which pore pressure oscillations penetrate into the rock under different conditions. We exclude the analytical solution *A*_AD_ from the further analysis because it was developed to represent laboratory conditions, but not the field cases. We include the typical range of properties of sandstones, granites, and shales based on the hydraulic properties indicated for these rock types (Brace 1980).

The attenuation curves exhibit an increasing linear trend for both geometries (red dashes—for 1D and black—for radial lines in Fig. 6), indicating that higher diffusivity leads to enhanced wave propagation. Similarly, an increase in the wave period *T* (or a decrease in frequency) shifts the curves toward greater distances for both geometries. This shift occurs without altering their slopes, implying that the slope of amplitude attenuation depends on the flow geometry rather than the wave's nature, i.e., the period *T*. Comparing *A*_*K*_ (red dashed line in Fig. 6) with *A*_R_ (black dashed line), we observe a more pronounced attenuation in the propagation of the pressure wave for the case of cylindrical flow *A*_*R*_, as anticipated by the previous results. This is evident through the curves' shift toward higher values and a steeper slope for the cylindrical geometry. For instance, considering *T* = 1 day, *A*_*K*_ reaches approximately 400 m, 0.08 m, and 2 m for Berea sandstone, Opalinus Clay, and Westerly granite, respectively, while *A*_*R*_ is limited to 90 m, 0.05 m, and 0.6 m for the same rocks. This results in a ratio of about 4 for sandstone, 1.6 for shale, and 3.33 for granite. In other words, the influence of the geometry becomes more pronounced with higher diffusivity of the material *D*.

**Fig. 7:**
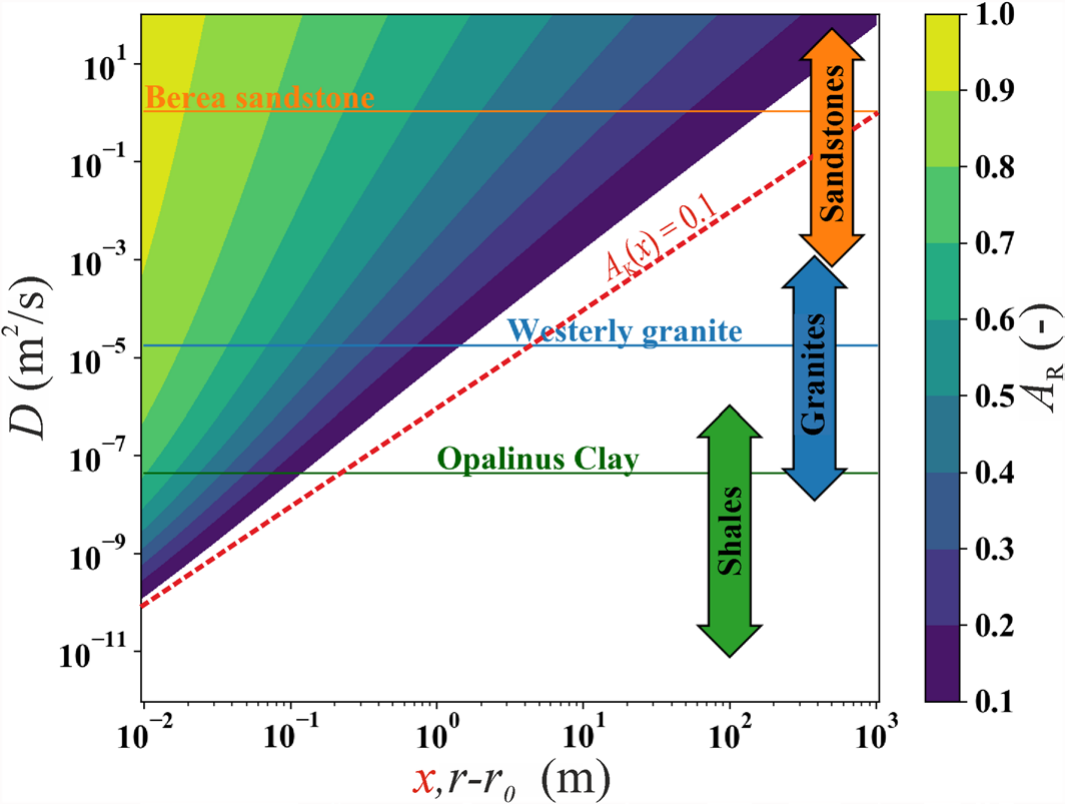
Contour plot of the amplitude ratio *A*_R_ as a function of radial distance from the source (*r*-*r*_0_) with a fixed period (*T* = 7 days). The line representing *A*_K_ = 0.1 for the one-dimensional distance from the source (*x*) is overlaid with the same period (*T*) depicted as a red dashed line. The horizontal orange, blue, and green lines correspond to the diffusivity (*D*) values used in this study for the sandstone, granite, and shale, while the diffusivity ranges for different rock types are based on the data from Brace (1980)

Figure 7 illustrates the contour plot of *A*_R_(*r*), representing the cylindrical diffusion of periodic pressure perturbation under constant stress for one week, and a threshold of 0.1, alongside the line *A*_K_(*x*) = 0.1 to visualize the difference between the two solutions. In the contour plot, it becomes evident that isolines with high *A*_R_ values (> 0.4) exhibit an exponential trend for the aquifer diffusivity when presented in a bi-logarithmic diagram, while for lower values, they have a linear trend. Conversely, the iso-*A*_K_ curves remain straight, as they follow a negative exponential function of *x* and the square root of 1/*D* (Eq. 5). This further accentuates the disparities between the two geometries and emphasizes the significance of selecting the right solutions according to the physics of the problem.

## Discussion

### Pore Pressure Response Affected by Rock Properties

The diffusivity *D*, defined as the ratio of hydraulic conductivity *κ* to specific storage *S*_s_, controls the amplitude attenuation and shift in the period of periodic signals in porous rock. High diffusivity is obtained by a combination of high stiffness (or low specific storage) and high permeability (and transmissivity). Notably, the significant difference in diffusivity between shales (*D* ~ 10^–11^ to 10^–6 ^m^2^/s) and sandstones (*D* ~ 10^–3^ to 10^2 ^m^2^/s) is mainly caused by their permeability contrast (Brace 1980). Additionally, the variation in diffusivity between shales and granites (*D* ~ 10^–8^ to 10^–3 ^m^2^/s) is a consequence of a significantly higher stiffness of the crystalline rock, as indicated in Table 1. It should be noted that the values we have assigned to the rock formations correspond to the intact material, rather than the entire rock mass. In the case of low-permeability rocks such as shales and granites, the hydraulic properties of the formation can be significantly affected by the presence of fractures (Brace 1980; van der Kamp 2001), which may result in a permeability enhancement by two to five orders of magnitude depending on their orientation, aperture, and sealing (Neuzil 1986; Rutqvist 2012; Bondarenko et al. 2022).

We report analytical solutions for specific geometries: *A*_K_ for one-dimensional flow (Eq. 5) and *A*_R_ for radial flow (Eq. 12). Comparing these with the more comprehensive numerical solution *A*_HM_ (Sect. 2.7.3), we find discrepancy across three geo-energy applications with highly diverse periodic signals and hosting rock characteristics (insets in Fig. 4 and Fig. 5). This disparity is principally attributed to neglecting mechanical controls on pressure diffusion by hydraulic solutions. While *A*_K_ and *A*_R_ assume uncoupled conditions, the coupled *A*_HM_ accounts for the instantaneous deformations of the rock caused by the oscillatory pore pressure perturbation and changes in the effective stresses. Besides, fluid accommodation and expulsion by porous rock involve various mechanisms, including the expansion/compaction of the solid frame, solid constituent, and the pore fluid characterized by their compressibility and weighted by the rock porosity. In unconsolidated aquifers where the rock body is highly compliant, it is common to neglect the compressibility of solid constituents when interpreting the in situ test data (Jacob 1950; Ferris 1952). However, in stiff aquifers, the compressibility of solid constituent is not negligible for the diffusion of periodic pore pressure, consistent with observations on tidal fluctuation by van der Kamp and Gale (1983).

A nearly constant amplitude attenuation that is observed away from a certain distance from the injection well is relevant when considering the hydro-mechanical coupling. For the design of the periodic CO_2_-rich water injection in caprock (CO_2_LPIE experiment), it is crucial to define the best location of the monitoring wells with respect to the injection well (Sciandra et al. 2022a; 2022b). Our analysis shows that the amplitude rapidly attenuates within a few tens of centimeters, suggesting that monitoring devices should be placed as close as possible to the injection well. However, some limitations exist related to the minimum distance between boreholes to avoid interferences and affections between the drillings and stability of the boreholes. Thus, the minimum required distance may be farther away from the injection well than the distance at which the amplitude attenuation becomes constant. Installing pressure sensors directly on the casing of the injection well could be a practical and efficient approach to monitor pressure fluctuations in the caprock-like material. Being aware of the hydro-mechanical rock behavior is essential when processing the monitoring data to avoid misinterpretations of diffusivity estimates.

### Pore Pressure Response Affected by Fluid Properties

The diffusivity parameter* D* is inversely proportional to fluid viscosity *μ* while decreasing with the decrease in fluid bulk modulus *K*_*f*_, which results in higher storativity. In this study, we consistently assumed water at 20 °C as the injected fluid in all test cases (Sect. 3) to put our emphasis on the role of injection parameters and rock characteristics. However, *μ* varies with temperature (e.g., decreases by a factor of 2 for water at 50 °C) and is relatively insensitive to pressure gradients. This dependence of viscosity on temperature is especially relevant in enhanced geothermal systems, which seek temperatures around 180 °C, representing a viscosity reduction of around one order of magnitude. Additionally, *K*_*f*_ changes with pressure, temperature, and salinity, but generally remains within the same order of magnitude (Osif 1988). While these observations apply to water, they may not be applicable when considering other fluids at different physical states.

In the context of energy storage and for CO_2_ Long-term Periodic Injection Experiment (CO_2_LPIE) scenarios (a and b in Fig. 1, respectively), the injected fluid could be H_2_, CH_4_, or CO_2_, in either liquid, gas, or supercritical phases. For instance, in the case of underground storage of H_2_ with an approximate compressibility of 70 MPa^−1^ and a viscosity of around 8.9·10^–6^ Pa·s (Zivar et al. 2021), the resulting diffusivity in Berea Sandstone is two orders of magnitude higher compared to the one of water (Table 1). Consequently, at a radial distance of 2 km and an annual period, the amplitude attenuation *A*_*R*_ (Eq. 12) increases from around 6% to about 23%.

Similarly, considering gaseous CO_2_ injection in CO_2_LPIE experiment, the compressibility becomes approximately 5 GPa^−1^, with a viscosity of around 3.6·10^–5^ Pa·s (Span and Wagner 1996), leading to a diffusivity of approximately 5.6·10^–7^ m^2^/s, one order of magnitude higher than the one with water (Table 1). Consequently, at a radial distance of 0.1 m and a period of a week, the amplitude ratio *A*_R_ would increase from about 25% to approximately 56%. Nonetheless, CO_2_ cannot easily penetrate shales like Opalinus Clay because of its nano-scale pore throat sizes and high entry pressures (Makhnenko et al. 2017). Therefore, the periodic signal of the injected gaseous CO_2_ is transmitted to the resident pore water, which is in essence the fluid that controls the amplitude attenuation and the shift in the periodic signal. CO_2_ will dissolve into the water and will advance by advection and diffusion, but not as free phase (Sciandra et al. 2022b).

A more complex scenario involves the examination of the interplay between two distinct fluids and their impact on the exposed rock. Two-phase flow in porous media is controlled by capillary pressure and relative permeability (Blunt et al. 1992). Laboratory investigations, when capillary displacements become dominant in the flow regime, are often conducted to determine these parameters (Bennion and Bachu 2008; Makhnenko et al. 2017). However, at the reservoir scale, the dynamics are predominantly governed by the interplay of viscous and buoyancy forces (Blunt et al. 1992). For analytical solutions, the gap between macroscopic flow and pore-scale displacements can be bridged through the utilization of the multiphase Darcy's law, as implemented in the numerical simulations (Vilarrasa and Makhnenko 2017; Kivi et al. 2022). In general, introduction of the non-wetting fluid (H_2_, CH_4_, or CO_2_ in different phases) results in the decrease in aqueous pore fluid bulk modulus and, thus, increase in the diffusivity value. An additional increase in diffusivity could be caused by the chemical effect, mostly pronounced for CO_2_ injection where acidic aqueous solution can significantly alter rock properties. The increase of the host rock permeability from a few percent in sandstones and shales to tens of percent in limestones have been reported for rock subject to in situ storage conditions (Vanorio et al. 2011; Alam et al. 2014; Kim et al. 2023; Kim and Makhnenko 2023). Finally, while our study focuses on pressure signals due to their direct relevance in estimating hydraulic diffusivity and permeability, other types of periodic signals—such as temperature fluctuations—can also offer valuable insights for rock characterization (Lord and Shulman 1967; Pathania et al. 2023). In geothermal applications, temperature signals are especially relevant, as thermal diffusivity provides complementary information on rock properties and fluid flow dynamics.

### Pore Pressure Response Affected by the Period

Our results exhibit an exponential decay of the amplitude ratio *A* on the characteristic length *λ*. Specifically, this characteristic length corresponds to the distance at which a one-dimensional amplitude attenuation of approximately 37% in the pressure wave is achieved, as described in Sect. 2.3. By defining the characteristic distance in terms of the period, we derive the expression *λ* = (*TD/2π*)^1/2^ (Sect. 2.1). Consequently, an increase in the period *T* causes the increase in *λ*, amplifying the diffusion of the pressure wave's amplitude. Therefore, when comparing different applications, short-period (or high-frequency) waves have a similar effect to fast pumping tests affecting a small portion of the aquifer, while long-period (or low-frequency) waves allow to characterize larger areas of the aquifer (Alcaraz et al. 2021). Accordingly, adopting a seasonal period, as in the case of energy storage (Lysyy et al. 2021), facilitates the diffusion of the pressure wave over kilometer-scale distances.

In the CO_2_ Long-term Periodic Injection Experiment (CO_2_LPIE), the optimal period remains a subject of ongoing discussion, spanning from daily to weekly scales, also being mindful of the relevant periods of the Earth tides. For example, focusing on the representative radial diffusion *A*_R_ (Sect. 2.5), this translates to an attenuation ranging from 0.008% for *T* = 1 day to 1.65% for *T* = 1 week at a distance of 0.3 m. This phenomenon has motivated the increase in the period, rather than elevation of the initial amplitude, particularly in laboratory settings where downstream wave pressure measurements may fall below the transducer resolution (Faulkner and Rutter 2000). Nonetheless, it is crucial to carry out an adequate number of cycles to accurately distinguish the influence of periodic injections at the measurement locations and allow for stacking analysis of small response signals. For instance, in the CO2CRC Otway field test, only three temporal cycles of pore pressure change were introduced, which proved to be insufficient to isolate their effects from other contributing factors (Ennis-King et al. 2017).

In the context of enhanced geothermal systems stimulation, the implementation of cyclic stimulation has emerged as a potential strategy to mitigate the risks associated with induced seismicity (Zang et al. 2013, 2019). For this reason, Hofmann et al. (2018) introduced an injection protocol comprising distinct cycles characterized by varying time scales: short-term cycles with periods on the order of minutes or less, medium-term cycles lasting for hours, and long-term cycles spanning a few days or weeks. The ultimate signal becomes a combination of these distinct time-scale cycles. By considering representative periods of 6 min, 1 h, and 1 day—similar to the Pohang enhanced geothermal system site in South Korea (Hofmann et al. 2019), the attenuation *A*_R_ obtained at a distance of 1 m equals 6·10^–9^%, 0.02%, and 7%, respectively. This outcome underscores the effectiveness of cyclic or soft stimulation in reducing the risk of induced seismicity because the amplitude vanishes within a few centimeters for periods on the order of minutes (as done in Pohang). This rapid attenuation of the periodic signal impedes the progressive degradation of fracture strength that is sought with this stimulation to reduce induced seismicity. Thus, the pressure perturbation in tight, stiff crystalline rock is very similar to constant pressure or periodic pressure injection with periods on the order of minutes.

### Accuracy of Analytical Models and Contribution of the Reservoir Deformation

The expansion (or compaction) of a rock caused by fluid injection/extraction can induce notable alterations in its storage behavior. As the rock undergoes expansion, there is a potential for pore spaces to open up, leading to increased effective porosity. This, in turn, can improve fluid flow pathways, ultimately enhancing the effective permeability and thus the rock transmissivity. Additionally, expansion increases the storativity by providing additional storage volume as the pore space expands. These effects are contingent on multiple factors, including the geological attributes of the rock, the characteristics of the fluids being injected or extracted, the rate of deformation, and the boundary conditions (Bear 2013). In particular, hydro-mechanical coupling becomes critically important in low-permeability rocks, such as shales and crystalline basements, by changing their storage behavior (De Simone and Carrera 2017). For example, small changes in porosity in these materials caused by the increase of the effective mean stress by just a few MPa can cause a decrease in permeability by more than an order of magnitude (Kim and Makhnenko 2020). The results obtained in this study show that the coupled hydro-mechanical effects on pressure amplitude attenuation, i.e., *A*_HM_—*A*_*H*_, during CO_2_ injection in underground rock laboratory and enhanced geothermal systems stimulation are twice as large as that calculated for energy storage applications in permeable rock (insets in Fig. 4 and Fig. 5).

### Applications of the Methods to Support Design and Monitoring Stages

**Fig. 8:**
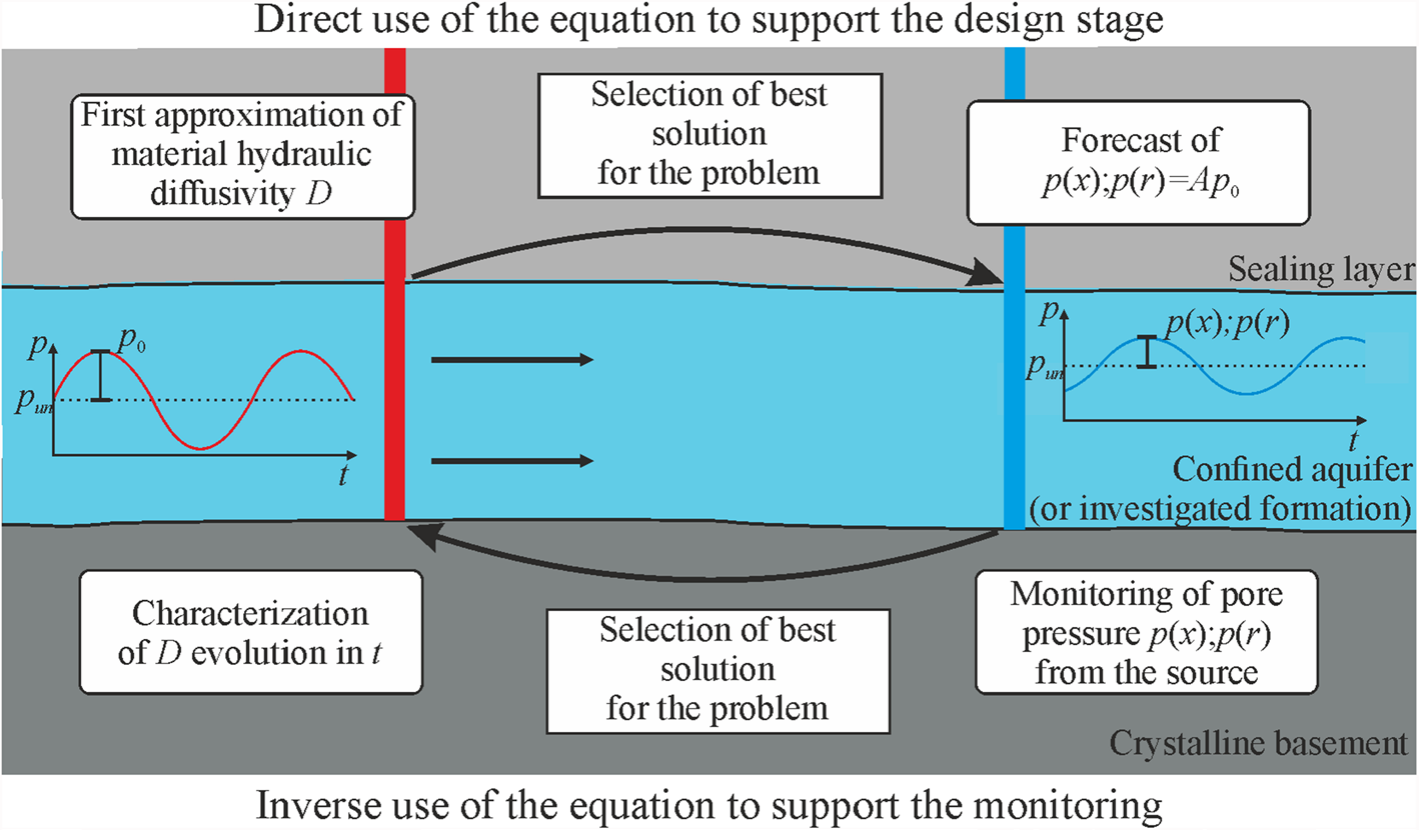
Description of “direct” and “indirect” use of the methods described in this article

We consider the application of the methodologies presented in this study in two ways: "direct" and "indirect" (Fig. 8). The direct utilization of the equations is principally subject to an initial estimation of material properties to calculate the diffusivity *D* and design the input signal characteristics including its period *T*. After selecting a suitable solution for the specific application, it is possible to project the signal attenuation at varying distances from the source. In the context of the indirect application of the equations, the focus shifts to employing monitoring data, including the amplitude at distance (*p*(*x*) or *p*(*r*)), to infer the properties of the rock mass between the source and the monitoring devices, which can be the injection/production well and the monitoring well, respectively (Fig. 8). If the theories are employed to interpret signals originating from passive sources, as in the case of natural Earth tides or those generated in laboratory setups featuring finite specimen lengths, additional parameters may be needed to properly represent the propagating pressure waves. The former requires knowledge of the source amplitude, phase, loading efficiency, and location (Merritt 2004; Alcaraz et al. 2021). For the latter, solutions rely on the storage behavior of the injection setups (Hsieh et al. 1981). As the need to know such parameters in purposefully-designed injections at field scales is eliminated, the focus can be directed toward the in situ characterization of the rock layers.

The obtained results show that the proper selection of a suitable and physically grounded model, within realistic boundary conditions, is of paramount importance for both direct and indirect applications (Fig. 8). Generally, during the design phase, numerical models hold greater relevance due to their capacity to replicate system deformations without temporal constraints for solution attainment. In contrast, the adoption of analytical solutions finds greater utility in aiding the monitoring stage for continuous characterization purposes. For example, in the case of CO_2_LPIE, in which CO_2_ dissolves into the water, reducing its pH and, as a result, causing mineral dissolution that enhances porosity and permeability and lowers stiffness, diffusivity continuously evolves and the monitoring of the periodic signal can provide valuable information on how CO_2_ injection affects rock properties with time. These solutions provide a rapid understanding, albeit approximate, of reservoir behavior evolution, facilitating quick insights into system dynamics, particularly when the computational speed is a crucial factor.

Selecting the appropriate period for signal analysis is crucial to ensure meaningful results tailored to the specific application. Practitioners should consider the following factors when determining the optimal period: characteristic timescale of the application, the material characterization before the injection/extraction, and the precision of the in situ instrumentation in monitoring the signal attenuation. They should select a period that balances measurable signal amplitude at the monitoring location with sufficient sensitivity to formation properties. Longer periods are suitable for high-permeability formations or larger monitoring distances, while shorter periods are better for low-permeability formations. The optimal period will also vary depending on the application. For instance, in energy storage, long periods may capture large-scale dynamics, while shorter periods may be more effective for near-wellbore diagnostics in geothermal systems. Field tests using a range of periods can help identify the signal that provides the clearest and most consistent signal, given site-specific conditions.

## Conclusions

In this study, we have investigated the feasibility of rock characterization through diverse analytical and numerical solutions for the interpretation of periodic pressure signals, employing both one-dimensional and cylindrical two-dimensional geometries. This investigation covers different rock types and various formation dimensions, ranging from kilometers for energy storage scenarios in sandstone to approximately half a meter for the CO_2_ Long-term Injection Experiment (CO_2_LPIE) in shale, and a few meters for enhanced geothermal systems stimulation in granite.

For any application, it is crucial to acknowledge that assuming the uncoupling of pore pressures and stress variations in rock introduces an element of error that warrants analysis. Our findings reveal that the relative error between the uncoupled problem and a simplified geometry is less than 3% for one-dimensional diffusion and less than 1.4% for radial diffusion. However, it is essential to recognize that more complex in situ scenarios, including geological inhomogeneity and anisotropy, hydraulic barriers, and potential leakage sources, may introduce more substantial errors that require careful examination for each specific case.

While hydro-mechanical numerical solutions account for multidimensional aquifer deformations, analytical solutions provide an immediate initial approximation of the problem, facilitating a better understanding of reservoir behavior evolution. This enables time prompt reaction to potentially unexpected events, safeguarding the integrity of the underground geo-energy projects.

## Figures and Tables

**Fig. 9:**
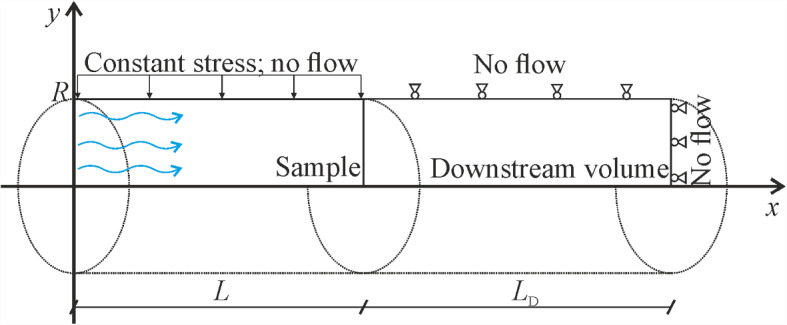
Axisymmetric numerical model (around *x*) with HM boundary conditions to reproduce Adachi and Detournay (1997) solution at the core scale

**Fig. 10:**
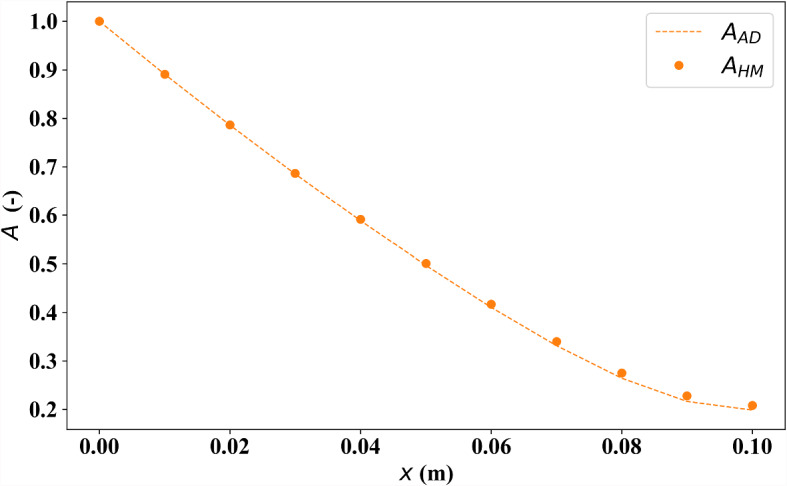
Amplitude ratio of Adachi and Detournay (1997) analytical solution A_AD_ and the numerical results A_HM_

## Data Availability

The datasets generated during and/or analyzed during the current study are available in the CSIC repository, which practices FAIR principles: https://doi.org/10.20350/digitalCSIC/15676

## Appendix 1

Validation of Adachi and Detournay (1997) solution at the core scale

To validate Adachi and Detournay’s (1997) solution at the core scale, we run a coupled HM numerical simulation reproducing the same conditions. The geometry represents an axisymmetric domain composed of a slender specimen of radius *R* = 0.03 m and length *L* = 0.1 m with a symmetry axis of *y* = 0 and a downstream compartment of the same radius to the rock specimen and length *L*_D_ = 0.106 m, resulting in a dead volume *V*_D_ = 3·10^–4^ m^3^ (Fig. 9).

We impose horizontal flow by applying a periodic pressure signal on the left-hand side of the specimen. The stress is constant on the lateral side of the specimen while displacement perpendicular to the downstream side is prevented (Fig. 9). In this way, the downstream volume is constant, given also the stiffness assigned to the material (Table

**Table 3:** Parameters to replicate the case study of Adachi and Detournay (1997) in our HM numerical simulation

Parameter	Sample	Downstream
*K* (GPa)	5.0	333.3
$K_{s}$(GPa)	33.0	222.2
*K*_*f*_ (GPa)	2.25	2.25
*ϕ* (−)	0.25	1.00
*k* (m^2^)	1.0·10^–14^	1.0·10^–9^
*B* (−)	0.60	1.00
*D* (m^2^/s)	2.90·10^–2^	1.67·10^3^

^3^). We solve for pressure diffusion into the specimen both numerically and analytically (Eqs. 4 and 7) using material properties listed in Table 3. The comparison of the analytical and numerical solutions shows almost perfect fitting (Fig. 10).

See Figs. 9 and 10, Table 3

## List of Symbols

*a*: The multiplicative constant of the Bessel function
*A*: Generic amplitude ratio
*A*_1_: Amplitude ratio used to calculate *x*_1_
*A*_2_: Amplitude ratio used to calculate *x*_2_
*A*_AD_: One-dimensional amplitude ratio in case of constant lateral stress
*A*_*F*_: Uncoupled 1D amplitude ratio for incompressible solid matrix
*A*_*H*_: The amplitude ratio of the hydraulic numerical problem
*A*_HM_: The amplitude ratio of the hydro-mechanical numerical problem
*A*_*K*_: Uncoupled 1D amplitude ratio
*A*_*m*_: One-dimensional amplitude ratio due to mechanical effect for constant lateral stress
*A*_*R*_: Uncoupled radial amplitude ratio
*b*: The multiplicative constant of the Bessel function
*B*: Skempton coefficient
*D*: Hydraulic diffusivity
*D*_*s*_: Hydraulic diffusivity for the incompressible solid matrix
*ϕ*: Effective rock porosity
*g*: Gravity acceleration
*h*: The thickness of the aquifer
*i*: Imaginary unit
*I*_0_: Bessel *I* function of order zero
*k*: Permeability
*κ*: Hydraulic conductivity
*K*: Bulk modulus of the rock skeleton under drained conditions
*K*_0_: Bessel *K* function of order zero
*K*_*f*_: Bulk modulus of pore fluid
*K*_*s*_: Bulk modulus of the solid phase including isolated pores
*L*: Length of the sample
*L*_*D*_: Length of the downstream
*μ*: Fluid viscosity
*N*: Coefficient defined to simplify the equations
*p*: Pore pressure
*p*(*r*): Pore pressure amplitude at distance *r* from the source
*p*(*x*): Pore pressure amplitude at distance *x* from the source
*p*_0_: Pore pressure amplitude at the source
*p*_un_: Undisturbed pore pressure
*θ*: Angular coordinate
*ρ*: Fluid density
*r*: Radial distance from the source
*r'*: Imaginary variable to calculate Bessel functions
*r*_0_: Well radius
*r*_1_: Representation distance for axisymmetric numerical models (taken equal to *x*_1_)
*r*_2_: Mesh dimension for axisymmetric numerical models (taken equal to *x*_2_)
*R*: Radius of the sample section
*S*_*s*_: Specific storage coefficient
*S*_*D*_: Storage coefficient of the downstream
*σ*_*kk*_: First invariant of the stress tensor
*σ*_*m*_: Total mean stress
*σ*_*xx*_: Total stress along diffusive direction
*t*: Time variable
*T*: Period at the source
*V*_*D*_: Volume of the downstream
*ω*: Frequency at the source
*x*: One-dimensional distance from the source
*x*_0_: Left boundary distance for plain strain numerical models (equal to *r*_0_)
*x*_1_: Representation distance for plain strain numerical models (evaluated from *A*_1_)
*x*_2_: Mesh dimension for plain strain numerical models (evaluated from *A*_2_)
*z*: Vertical coordinate
